# An Electronic Clinical Decision Support System for the Assessment and Management of Suicidality in Primary Care: Protocol for a Mixed-Methods Study

**DOI:** 10.2196/11135

**Published:** 2018-12-07

**Authors:** Matthew Horrocks, Maria Michail, Aimee Aubeeluck, Nicola Wright, Richard Morriss

**Affiliations:** 1 Institute of Mental Health Division of Psychiatry and Applied Psychology, School of Medicine University of Nottingham Nottingham United Kingdom; 2 School of Health Sciences University of Nottingham Nottingham United Kingdom; 3 Institute for Mental Health School of Psychology University of Birmingham Birmingham United Kingdom

**Keywords:** suicide, primary care, general practitioner, clinical decision support system

## Abstract

**Background:**

Suicide is a global public health concern, but it is preventable. Increased contact with primary care before the suicide or attempted suicide raises opportunities for intervention and prevention. However, suicide assessment and management are areas that many general practitioners (GPs) find particularly challenging. Previous research has indicated significant variability in how GPs understand, operationalize, and assess suicide risk, which subsequently has an impact on clinical decision making. Clinical decision support systems (CDSS) have been widely implemented across different health care settings, including primary care to support practitioners in clinical decision making. A CDSS may reduce inconsistencies in the identification, assessment, and management of suicide risk by GPs by guiding them through the consultation and generating a risk assessment plan that can be shared with a service user or with specialized mental health services.

**Objective:**

Our aim is to co-develop and test with end users (eg, GPs, primary care attendees, mental health professionals) an electronic clinical decision support system (e-CDSS) to support GPs in the identification, assessment, and management of suicidality in primary care.

**Methods:**

Ours is an ongoing embedded mixed-methods study with four phases: (1) qualitative interviews with GPs to explore their views on the content, format, and use of the e-CDSS, as well as consultation with two service-user advisory groups (people aged ≤25 and people aged ≥25) to inform the content of the e-CDSS including phrasing of items and clarity; (2) participatory co-production workshops with GPs, service users, and clinical experts in suicidality to determine the content and format of the e-CDDS; gain consensus of the relevance of items; establish content validity and identify pathways to implementation, using the Consolidated Framework for Implementation Research; (3) building the e-CDSS so that it guides the GP through a consultation; and (4) usability testing of the e-CDSS with GPs and service users in one primary care practice involving a nonlive and a live stage.

**Results:**

The study was funded for four years, to take place between 2015 and 2019, and is currently completing phase 4 data collection. The first results are expected to be submitted for publication in June 2019. The findings will enable us to evaluate the feasibility, acceptability, and usability of a suicide-specific, electronic, guided decision support system in primary care.

**Conclusions:**

This study will be the first to explore the feasibility, acceptability, and usability of an electronic, guided decision support system for use in primary care consultations for the improved assessment and management of suicidality.

**International Registered Report Identifier (IRRID):**

RR1-10.2196/11135

## Introduction

Suicide is a global public health concern costing the lives of over 800,000 people per year [1]. It is among the three leading causes of death in those aged 15-44 and the second leading cause of death among children and young people aged 15-29 [1]. Among the strongest risk factors for suicide are history of suicide attempts, mental illness, and self-harm [2]. The likelihood of suicide may be reduced where health professionals and service users openly and compassionately identify and collaboratively address suicide risk. Research shows increased contact with health professionals and in particular, general practitioners (GPs), in the months prior to a suicide or an attempted suicide [3-5]. Contact rates range from 60%-83% in the 12-month period before suicide [4-6], and 45% of those who die by suicide are likely to have had contact with a primary care provider within 4 weeks of suicide [3]. Primary care is often both the first and last health care contact for those who take their life [7]. These high rates of pre-suicide contact suggest that primary care services are well placed to identify early, assess, and mitigate risk of suicide. Primary care is, therefore, considered an appropriate context to develop suicide prevention initiatives [8].

Suicide risk assessment and management, however, is an area that most GPs find particularly challenging, despite being a common feature of their work. In a typical morning or afternoon primary care surgery in the United Kingdom, at least one new case of depression out of 20 patients requires suicide risk assessment [9]. Organizational barriers including time constraints and a heavy workload coupled with a lack of specialist clinical skills and insufficient mental health training have been identified by GPs as significant barriers to the assessment and management of suicidal presentations [10-12]. A recent study by Michail et al [11] has identified significant variability in how GPs understand and operationalize risk, which subsequently has an impact on clinical decision making. GPs may be more likely to inquire about suicide risk following recognition of clinical features associated with depression, psychosis, or long-term physical health problems [13], yet evidence shows that depression is not systematically detected and managed by GPs [14,15]. This variability in clinical decision-making processes may be because practitioners tend to develop “mindlines” or heuristics linking certain risk factors with eventual outcomes [16,17]. Such cognitive devices may be developed by practitioners to aid information gathering and clinical decision making in time-pressured contexts. Heuristic-based decision making enables a rapid problem-solving approach to fast track a diagnosis or clinical decision; this is sometimes referred to as a “pattern matching” approach to clinical reasoning [18]. However, these cognitive shortcuts employed by practitioners could prove to be problematic. If allowed to become automatic and unconscious, they could lead to misdiagnosis and poor patient experience [19]. Heuristics and “gut-hunches” may play an important role in determining when clinicians inquire about suicide risk and influencing situations where practitioners fail to identify suicide potential [20].

Clinical decision support systems (CDSS) are “any electronic system designed to aid directly in clinical decision making, in which characteristics of individual patients are used to generate patient specific assessments or recommendations that are then presented to clinicians for consideration” [21]. A recent systematic review identified three categories of CDSS used across different health care settings, including primary care [22]. These include decision prompts, information retrieval systems, and bibliographic databases. All three types of CDSS have been shown to be positively associated with improved health care delivery including enhanced clinical decision making, supporting accurate diagnosis, and improving standards of chronic disease management and preventative care [22]. One of the added benefits of CDSS software is the “cognitive forcing function,” which may temporarily prompt the GP to switch from heuristic based to analytical decision making [23].

A suicide-specific electronic CDSS (e-CDSS) could address some of the aforementioned barriers to suicide assessment and management in primary care [11] by guiding GPs through the clinical risk assessment at the time of the consultation. Although there are several evidence-based suicide prevention training programs, for example, Applied Suicide Intervention Skills Training, and Skills-Based Training On Risk Management (STORM) for suicide prevention demonstrating sustained improvements in knowledge, skills, and attitudes [23-26], these do not address the challenges to assessment and management of suicidality in primary care, such as lack of guidance during the consultation and support in clinical decision making [11]. Suicide prevention training programs for GPs specifically have produced ambiguous results as many of these are provided to health professionals at population levels, rather than targeting GPs at their work place in primary care [27].

On the contrary, a suicide-specific e-CDSS could provide a standardized method of recording risk history and flagging ongoing social circumstances or risk factors [11], thus, facilitating appropriate management options. Most importantly, it could save the GP work and time by generating a risk assessment and management plan that can be shared with a service user and carer(s) or with specialized mental health services. Emerging evidence suggests that technology-based suicide prevention developments can assist clinicians with the identification and management of suicide risk, by providing clinical decision support [28]. However, this is still an underexplored area.

The aim of this study is to co-develop and test with end users (eg, GPs, primary care attendees, mental health professionals) an e-CDSS to support GPs in the identification, assessment, and management of suicidality in primary care.

## Methods

### Design

**Figure 1:**
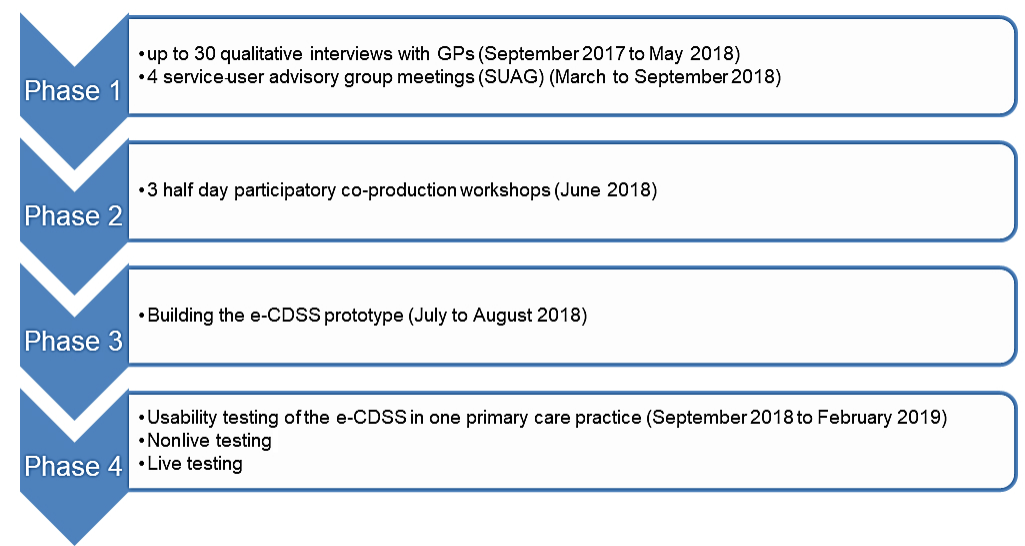
Study research design. e-CDSS: electronic clinical decision support; GP: general practitioner.

This is an embedded mixed-methods study incorporating a quantitative strand within a broader qualitative design (Figure 1). This design allows for bringing together insights from the different stages of the study to give a comprehensive approach to content development and initial evaluation of the e-CDSS in practices. The study will take place in the East Midlands, United Kingdom, between September 2017 and February 2019. The study received ethics approval by East Midlands - Nottingham 1 Research Ethics Committee (17/EM/0317).

### Sample and Recruitment

#### Phase 1

##### Qualitative Interviews With General Practitioners

GPs working in National Health Services (NHS) primary care practices across the East Midlands will be invited to take part in an individual face-to-face qualitative, semistructured, audiotaped interview with one researcher (MH) to explore their experiences of assessing suicide risk in primary care as well as their clinical reasoning and decision making about risk management. The interviews will also explore GPs’ views on the content, format, and use of the e-CDSS during consultation with at-risk individuals.

For Phase 1, up to 30 GPs will be recruited from primary care practices across the East Midlands region. The GP cluster leads, the mental health lead, GPs, or mental health commissioners across the various clinical commissioning groups in East Midlands will be initially approached to discuss the study and recruitment procedure. An invitation letter will be cascaded by email to all GPs by either the mental health lead GPs/commissioners or cluster leads, to inform potential participants about the study and encourage them to participate. Interviews with GPs will take place at times and locations convenient to them. For Phase 2, a purposive sample of GPs (ie, age, gender, ethnicity, and years of experience) will be drawn from Phase 1 and invited to attend the co-production workshops.

##### Service-User Advisory Group

To inform the development and design of the e-CDSS, two service-user advisory groups (SUAG) (people aged ≤25 and people aged ≥25) will be convened and meet (separately) four times between March 2018 and September 2018. The aim of this consultation would be to discuss potential items for inclusion in the e-CDSS, including phrasing and clarity, and to gauge the advisory groups’ view of whether proposed items might facilitate further disclosure or hinder concealment of suicide-related information.

For Phase 1, up to 10 participants aged 14-65 registered with a GP in Nottingham City or Nottinghamshire County will be recruited from various sources. These include primary care practices, third sector organizations, charities, self-help groups, existing public and patient involvement networks (eg, Collaboration for Leadership in Applied Health Research and Care [East Midlands] Patient and Public Involvement work stream), and social media. Participants would have to have a history of being through a GP consultation where suicidal thoughts, feelings, or behaviors had been discussed. For young people under the age of 16, participation is conditional on parental/guardian consent. For Phase 2, a purposive sample of service users (eg, age, gender, ethnicity) will be drawn from Phase 1 and invited to attend the co-production workshops.

#### Phase 2

##### Co-Production Workshops

A group of experts and health care professionals including GPs and service users from Phase 1 (drawn based on age, gender, and ethnicity) as well as clinical experts in suicidality will be invited to attend three half-day participatory co-production workshops. In the first co-production workshop, the research team will present the expert group with a list of questions and prompts extracted from (1) previously published assessment scales for suicide and self-harm, and (2) GP data during Phase 1, for inclusion in the e-CDSS. Using a modified Delphi approach, the expert group will individually rank these items according to their perceived relevance for assessment of self-harm and suicidality using a 4-point Likert scale (1=not relevant, 2=somewhat relevant, 3=quite relevant, 4=highly relevant). A prespecified consensus margin (≥65%-70%) used by previous similar studies [29] will be used to determine inclusion of items in the e-CDSS. In the second co-production workshop, the research team will redistribute those items within the prespecified consensus margin and ask participants to rank these items again according to their perceived relevance for assessment of self-harm and suicidality using the same 4-point Likert scale. After finalizing the items for inclusion in the e-CDSS, participants will be asked to complete the Content Validity Index (CVI) questionnaire to establish content validity (>0.80) [30]. This will then be followed by group discussion to reaffirm the face and content validity of the final items and to gain endorsement of items for inclusion in the prototype e-CDSS. This reconciliation of the data-driven methodological approach with the situated knowledge and perspective of GPs, people with lived experience, and suicide prevention experts is important to ensure goodness of fit of the prototype with end users and within the primary care context. The aim of the third co-production workshop would be to identify pathways to implementation as well as barriers and facilitators of adopting the e-CDSS in routine practice, using the Consolidated Framework for Implementation Research [31]. The aim would be to create a “change map,” that is, a graphical depiction of the pathway to long-term implementation of the e-CDSS.

#### Phase 3

##### Building the e-CDSS

Findings from Phase 1 and 2 will inform the most appropriate solution for an e-CDSS. The tool will be built by PRIMIS, a business unit of the University of Nottingham that specializes in building software to interface with and interrogate primary care information systems [32]. The tool will use clinical and informatics expertise alongside stakeholder requirements to present an option appraisal for a solution. Where possible, solutions will be based on existing Clinical Terms and Read Codes (ie, mandatory clinical coding systems for GP information technology systems). PRIMIS will account for current GP recording practices and the results of previous literature reviews in the field. A prototype of the tool will be developed in either The Phoenix Partnership (TPP) SystmOne or Egton Medical Information System (EMIS Web). The tool will take the form of a clinical system “protocol” (decision support algorithm) that will be response driven, that is, entries to the protocol will guide the user to the next appropriate stage.

#### Phase 4

##### Usability Testing of e-CDSS

An iterative evaluation of the e-CDSS prototype over a 6-month period will be conducted using an established theoretical and methodological framework [33] to refine the content of the final prototype, assess its usability, and provide the basis for initial evaluation of the e-CDSS in practice. The usability testing will be carried out in one primary care practice, and all GPs within this practice will be invited to participate. Usability testing will involve a nonlive and a live stage. Nonlive testing will involve GPs entering data into the e-CDSS in relation to simulated suicidal consultations based on clinical vignettes and completing a think-aloud exercise (“cognitive walk-through”) in which they will be asked to describe possible next questions and lines of inquiry following on from prompts and items within the e-CDSS. GPs will be asked to complete a brief evaluation questionnaire (System Usability Scale [SUS]) [34] relating to the usability and function of the e-CDSS. Following “go-live” of the system, GPs will be asked to use the prototype e-CDSS during live patient consultations if and when appropriate (eg, during scheduled mental health clinics). Following the use of the e-CDSS, GPs will be asked to complete the SUS as well as a short survey questionnaire about the overall relevance of the e-CDSS to suicidal consultations, its impact on clinical decision and management, its impact on workflow, as well as adoption and acceptability.

Usability testing will take place in one practice (Nottingham City or Nottinghamshire County), and all GPs employed within the practice will be eligible to participate. The recruitment of the practice will be based on convenience sampling based on accessibility and expressions of interest.

At this stage, the study will not be seeking feedback from primary care service users who have been in a consultation where the prototype e-CDSS is used, since the aim of the current study is to design the content for and to build an e-CDSS to support GPs in the identification, assessment, and management of suicidality in primary care. The current study will seek to investigate the compatibility of the e-CDSS with GPs’ consultation styles, the impact and integration into GP workflow, and its acceptability and feasibility to GPs. Further research is planned to formally investigate service user satisfaction within consultations in which the tool is used.

##### Feasibility, Usability, and Acceptability Criteria for Success for the e-CDSS

A set of predetermined criteria, in line with previous studies [35,36], will be used to assess feasibility, usability, and acceptability of the e-CDSS (Table 1). These criteria will be measured using data from co-production workshops (Phase 2), as well as the SUS and the survey questionnaire data GPs provide during usability testing (Phase 4). If the e-CDSS prototype does not reach the criteria for success, it will be refined according to the users’ needs and retested by GPs until it is fully adapted to their requirements.

**Table 1:** Electronic clinical decision support (e-CDSS) feasibility, usability, and acceptability criteria for success.

Measure	Criteria	Study phase
Content validity	>0.80 on the Content Validity Inventory	Phase 2: Co-production workshops
Usability	≥70 on the System Usability Scale	Phase 4: Usability testing (nonlive and live stage)
Adoption/Acceptance	Frequency of use—evidence that the e-CDSS is used by general practitioners at least once in a surgery with any new patient with depression, or new or severe mental health problems	Phase 4: Survey questionnaire (free-text response plus Likert Scales)
Feasibility and relevance in practice	GP reports and feedback on satisfaction with the e-CDSS; perceived barriers; ideas for improvement; ideas for further utilization; impact on workflow; impact on content of consultation; and impact of management (eg, referral rates, prescription rates)	Phase 4: Survey questionnaire (free-text response plus Likert Scales)

### Measures

#### Content Validity Index

The Content Validity Index (CVI) [30] is a widely used method of quantifying content validity for questionnaires with multiple items [30]. The CVI captures interrater agreement, through a standardized approach to computing agreement for each proposed item for inclusion in the questionnaire (or in this case the e-CDSS), as well as for the overall questionnaire. Participants are asked to rate each proposed item on an ordinal scale from 1=not relevant to 4=highly relevant. For each item, the number of ratings assigning 3 or 4 is then divided by the number of respondents to provide the proportion agreeing on the relevance of the item. The average score across each item of the e-CDSS will then also be calculated to generate a global content validity score. Scores of 0.80 are often considered the lower acceptable limits [30]. Application of the CVI will allow quantification of interrater agreement and be followed by group discussion to finalize the items for inclusion in the e-CDSS.

#### System Usability Scale

The SUS [34] will be administered to GPs during Phase 4 to measure the usability of the e-CDSS. This has been used extensively to assess the usability of a wide range of products and services including hardware and software, mobile devices, websites, and apps [37]. The SUS comprises 10 statements rated on a 5-point Likert scale (strongly agree to strongly disagree), recording respondents’ views of the usability of the e-CDSS. Total scores for the SUS range from 0-100. Published guidance [37] suggests that products with adequate usability will score above ≥70.

#### Survey Questionnaire

A short survey questionnaire for completion by GPs at Phase 4 will be developed based on data from GP interviews and SUAG meetings (Phase 1) as well as relevant literature [11] to assess the overall relevance of the e-CDSS to consultations in relation to suicide presentations, its impact on workflow, adoption, and acceptance as well as its impact on the content of the consultation and clinical outcomes (eg, referral and prescription rates). The survey questionnaire will use a combination of Likert scales and free-text response. Quantitative items will be calculated to provide total scores, mean scores, and variance scores for each item across participants to provide an indication of acceptability of the e-CDSS to GPs in relation to clinical appropriateness, contextual appropriateness, and quality of actionable decision support. High variance might be seen for some items where practices vary a lot in context and less so on other items.

### Data Analysis

#### Phase 1

GP qualitative interviews will be audio recorded and fully transcribed removing any identifiable data to preserve participant anonymity. Data will be analyzed using thematic [38] and content analysis [39]. Thematic analysis will allow the examination and recording of themes (patterns) within the GP data in relation to challenges in assessing and managing suicide risk in primary care, barriers and drivers for the use of the e-CDSS, and potential items and prompts for inclusion in the e-CDSS. Content analysis will be used to provide frequencies of coded themes including frequencies of those items and prompts mentioned by GPs during the interviews. NVivo 11 software [40] will be used to facilitate data analysis. Field notes kept during the SUAG group meetings will be presented in narrative form [41].

#### Phase 2

Statistical analysis will be descriptive (IBM SPSS 24.0). Consensus agreement will be calculated using counts (n) and proportions (%) for a median relevance rating of ≥3.25. Regarding the CVI, for each item the number of ratings assigning 3 or 4 will be divided by the number of respondents to provide the proportion agreeing with the relevance of the item. The average score across each item of the e-CDSS will then be calculated to generate a global content validity score. Scores of >0.80 are considered acceptable [30].

#### Phase 4

Consent, recruitment, and retention rates of GPs participating in the usability testing will be calculated. SUS item scores will be summed to obtain total scores. Total scores of ≥70 indicate adequate usability [37]. For the survey questionnaire assessing adoption, acceptability, relevance, and impact on management and workflow, we will present the mean, variance or standard deviations, and 95% confidence intervals for normally distributed variables, the median and interquartile range for skewed variables, and the frequency and proportion for categorical variables.

## Results

The study was funded for 4 years, to take place between 2015 and 2019, and is currently completing phase 4 data collection. Enrollment to Phase 1 (GP interviews) was completed in May 2018, with 30 GPs interviewed, and 6 people with lived experience taking part in SUAGs. Phase 2 (co-production workshops) was completed in June 2018, with a total of 24 participants taking part (ie, GPs, service users, commissioners, mental health clinicians, and subject matter experts in suicide prevention). Phase 3 (building the e-CDSS is currently underway) and Phase 4 (usability testing of the e-CDSS) is scheduled to take place between autumn 2018 and early 2019. Data analysis will be ongoing throughout Phase 4, with the first results expected to be submitted for publication in June 2019.

## Discussion

### Principal Considerations

Suicide is a major public health issue, but it is preventable. As the majority of pre-suicide contact takes place in primary care, GPs have an increasingly important role in the early identification, assessment, and management of suicide risk. This research will lead to the development of an evidence-based, electronic, guided decision support tool for use in primary care for the improved assessment and management of suicidality. The end product will be the output of collaboration and co-production between the research team, health informatics experts, and key stakeholders including primary care practitioners and service users. This collaborative approach will facilitate the implementation and uptake of the tool, which is a potential gain of this research. In addition, this research is expected to raise awareness, improve education of GPs about suicide, and promote best practice in assessing and managing risk. If the feasibility, acceptability, and usability of the e-CDSS are established in this study, then further research would be needed to establish its effectiveness and efficiency in routine primary care consultations. A pilot, cluster (practice level) randomized controlled trial of e-CDSS versus usual care would examine whether the use of e-CDSS leads to improved skills and capacity of GPs to manage suicidal behavior. The primary outcome would be assessed using the Suicide Intervention Response Inventory-2 [42] at baseline, postintervention, and 6 months follow-up. Secondary outcomes would include self-reported preparedness measures [43]; GPs’ attitudes (Attitudes Towards Suicide [44]) and confidence (using the 5-item STORM confidence in the assessment and management of suicidal people scale) [24]; service user satisfaction using qualitative interviews; and cost-effectiveness. If the effectiveness and cost-effectiveness of the e-CDSS is established, then this research could lead to improved assessment and management of suicidality in primary care and better patient experience of primary care mental health services.

### Conclusions

This study will be the first to explore the feasibility, acceptability, and usability of an electronic, guided decision support system for use in primary care consultations for the improved assessment and management of suicidality. A CDSS may reduce inconsistencies in the identification, assessment, and management of suicide risk by GPs by guiding them through the consultation and generating a risk assessment plan that can be shared with a service user or with specialized mental health services.

## Appendix

Multimedia Appendix 1 Peer-reviewer report from the CLAHRC-EM scientific committee.

## Abbreviations

CVI: Content Validity Index
e-CDSS: electronic clinical decision support
GP: general practitioner
NHS: National Health Services
STORM: Skills-Based Training On Risk Management
SUAG: service-user advisory group
SUS: System Usability Scale
