# Supplementary material for: An Electronic Clinical Decision Support System for the Assessment and Management of Suicidality in Primary Care: Protocol for a Mixed-Methods Study
Source: JMIR Res Protoc. 2018 Dec 7;7(12):e11135. doi: 10.2196/11135 (PMC6303673; doi:10.2196/11135)
Supplement: Multimedia Appendix 1 [file resprot_v7i11e11135_app1.pdf]

Reviewer 1

Project Name: The development and evaluation of an electronic suicide risk screening tool for use in in primary care

Criteria	Comments
Likely to bring benefits to patients and the public	No
Have strong interest and engagement from services and commissioners across the East Midlands	The application does name a number of clinical commissioning groups and NHS Trusts.
Involve co-production between researchers and those expected to use or benefit from the findings	In the current application there is no PPI involvement although if funded the application mentions that the public will be involved at a number of levels.
Are likely to be implemented by CLAHRC EM Partners	Unsure
Address important issues on chronic disease and public health	Yes
Be led by experienced research teams with a strong track record of delivery in applied health research	One of the applicants has a reasonable track record in research publications.
The project represents excellent value-for money.	No
Have an impact on patient outcomes within 3-5 years<br><br>Deliver significant reductions in service demand, improved service quality and/or efficiency gains<br><br>Reduce disparities in health between socially	Unlikely to have an impact in such a short period of time.<br><br>Unlikely.

disadvantaged and advantaged groups<br><br>Include plans for the adoption of findings, in collaboration with the EM AHSN<br><br>Lead to increased capacity to conduct applied health research (e.g. through doctoral studentships)<br><br>Involve collaborations with commercial organisations and other NIHR funded infrastructure	This section is well reported including the use of knowledge brokers.<br><br>There is no indication regarding this.					
**Do you recommend that NIHR CLAHRC EM fund this Project?**	**Yes**		**Maybe**		**No**	
**Please give the key reasons for your recommendation?**	There are major concerns regarding this proposal. One of the concerns is the huge amount being charged by PRIMIS as I am aware of other studies being conducted by PRIMIS at much lower costs. The background of the application gives rationale for conducting this study with a number of references. However a reference list has not been included. The references that they have cited do not in fact refer to studies that have developed computerised screening tools for risk of suicide. The key aim of the study is to develop an electronic suicide screening tool. Although methodologically it is clear that the applicants are not developing a new tool. For the development of a new tool one would need to have an outcome of suicide or indeed some validity of the primary outcome. Analysis plan would include development of risk score using ROC methodology which again is not included in the application. The authors state that they will work with PRIMIS to develop a computerised risk assessment tool. However one could develop a risk assessment tool using the CPRD database which I am not aware has been attempted previously.					

Reviewer No2

Project Name: The development and evaluation of an electronic suicide risk screening tool for use in primary care.

Criteria	Comments
Likely to bring benefits to patients and the public	Yes – fits with the national strategy 2012 and local suicide prevention plan (City CCG).
Have strong interest and engagement from services and commissioners across the East Midlands	Identifies 'county CCG' this is an out of date term, therefore cannot identify which CCG is involved or if multiple CCGs. Includes local NHS providers, mental health.
Involve co-production between researchers and those expected to use or benefit from the findings	Focus solely on identification through GP using a risk profile.<br>As 30% known/make contact with GP (research identify 2002 date as 45%) numbers of suicides increasing.
Are likely to be implemented by CLAHRC EM Partners	
Address important issues on chronic disease and public health	Fits with national strategy and local plans. Identified as a priority and in patient safety collaboration.
Be led by experienced research teams with a strong track record of delivery in applied health research	Cannot comment re GP involvement and experience but supported by experienced researchers.
The project represents excellent value-for money.	50% costs relate to salary costs.<br>Matched funding £2,700 from CCG.
Have an impact on patient outcomes within 3-5 years<br><br>Deliver significant reductions in service demand, improved service quality and/or efficiency gains<br><br>Reduce disparities in health between socially	Will lead to further research therefore impact may not be seen until 5 years plus. However education ,ay improve.<br>The tool is to be tested, the approach not proven in England; however Primary Care identified as a focus.<br><br>Suicide is linked to disparities in health and social groups.

<table>
<tr><td colspan="1">disadvantaged and advantaged groups

Include plans for the adoption of findings, in collaboration with the EM AHSN

Lead to increased capacity to conduct applied health research (e.g. through doctoral studentships)

Involve collaborations with commercial organisations and other NIHR funded infrastructure</td><td colspan="6">Use of knowledge brokers.

Will lead to multi-centre study and application for NIHR funding,</td></tr>
<tr><td>Do you recommend that NIHR CLAHRC EM fund this Project?</td><td>Yes</td><td>✓</td><td>Mayb e</td><td></td><td>No</td><td></td></tr>
<tr><td>Please give the key reasons for your recommendation?</td><td colspan="6">This is a national strategy.
Recent reports (2014) demonstrated urgent consideration/action to prevent suicide out if the hospital setting, identifying GP as one area of focus.
A screening tool is one approach but not yet tested in this area.
May lead to additional benefits of increased awareness and education locally.
Essentially will require further, large scale, research.
PSC, identified as a key priority.
Would increase profile of locality.</td></tr>
</table>

Reviewer 3

Project Name: The development and evaluation of an electronic suicide risk screening tool for use in in primary care

Criteria	Comments
Likely to bring benefits to patients and the public	Project is likely to bring benefits to public and patients by development of new electronic tool for assessment of risk of suicide in primary care.
Have strong interest and engagement from services and commissioners across the East Midlands	The topic selected for this piece of research will attract strong interest from service users and commissioners
Involve co-production between researchers and those expected to use or benefit from the findings	The proposed plan to involve co-production is robust
Are likely to be implemented by CLAHRC EM Partners	Yes, it's likely to be implemented by partners of EM CLAHRC
Address important issues on chronic disease and public health	Correct identification of suicide risk is important issue and the proposed project aim to address this.
Be led by experienced research teams with a strong track record of delivery in applied health research	The given profile of research team fits nicely in to feasibility of this project
The project represents excellent value-for money.	Yes
Have an impact on patient outcomes within 3-5 years<br><br>Deliver significant reductions in service demand, improved service quality and/or efficiency gains<br><br>Reduce disparities in health between socially	Given research proposal will definitely have impact on clinical practice in primary care in identifying the suicide risk in vulnerable patient correctly and referring patient in time but at the same time reducing the improper referrals to secondary care on suspicion of suicide risk.<br>The proposal has plans for adoption of findings in collaboration with EM AHSN and also has a potential to lead to development of capacity to conduct applied health research.

<table>
<tr><td>disadvantaged and advantaged groups

Include plans for the adoption of findings, in collaboration with the EM AHSN

Lead to increased capacity to conduct applied health research (e.g. through doctoral studentships)

Involve collaborations with commercial organisations and other NIHR funded infrastructure</td><td colspan="6"></td></tr>
<tr><td>Do you recommend that NIHR CLAHRC EM fund this Project?</td><td>Yes</td><td>x</td><td>Mayb e</td><td></td><td>No</td><td></td></tr>
<tr><td>Please give the key reasons for your recommendation?</td><td colspan="6">The chosen topic is quite relevant to our day to day clinical practice and will attract service users and commissioners. As rightly described, majority of work on risk estimation for suicide is done outside of UK and there is need to produce local evidence to direct our clinical practice.</td></tr>
</table>
